# Supplementary material for: Endoscopic transpapillary gallbladder drainage for the management of acute calculus cholecystitis patients unfit for urgent cholecystectomy
Source: PLoS One. 2020 Oct 9;15(10):e0240219. doi: 10.1371/journal.pone.0240219 (PMC7546490; doi:10.1371/journal.pone.0240219)
Supplement: S1 Table — (DOCX) [file pone.0240219.s001.docx]

**S1 Table. Baseline characteristics of patients with successful SpyDS assisted cystic duct cannulation.**

| **Case** | **Age/Sex** | **Co-morbid condition** | **ASA class** | **Diagnosis** | **Visible cystic duct by cholangiography** |
| --- | --- | --- | --- | --- | --- |
| 1 | 87/F | Complete AV block with pacemaker, cerebral infarction with left hemiparesis, HTN, DM | III | Presence of CBD stone with ACC | No |
| 2 | 80/F | Dementia, heart failure, HTN | III | Presence of CBD stone with ACC | No |
| 3 | 83/F | Parkinson’s disease, dilated cardiomyopathy, HTN, DM | III | Presence of CBD stone with ACC | No |
| 4 | 87/F | Dementia, cerebral infarction, HTN, DM, CKD | III | Presence of CBD stone with ACC | No |
| 5 | 74/F | Heart failure, mitral valve replacement, intracranial hemorrhage | III | Presence of CBD stone with ACC | No |
| 6 | 70/M | ESRD with hemodialysis, DM, HTN | III | Presence of CBD stone with ACC | No |
| 7 | 79/F | ESRD with hemodialysis, Parkinson’s disease, HTN | III | Presence of CBD stone with ACC | No |
| 8 | 81/F | DM, HTN, dementia multiple myeloma | III | Presence of CBD stone with ACC | No |

*SpyDS Spyglass^TM^ DS Direct Visualization system****;*** *ASA, American Society of Anesthesiologists, CBD common bile duct, AV atrioventricular , ACC acute calculus cholecystitis, HTN hypertension, DM diabetes mellitus, CKD chronic kidney disease, ESRD end-stage renal disease.*
