# Supplementary material for: Endoscopic transpapillary gallbladder drainage for the management of acute calculus cholecystitis patients unfit for urgent cholecystectomy
Source: PLoS One. 2020 Oct 9;15(10):e0240219. doi: 10.1371/journal.pone.0240219 (PMC7546490; doi:10.1371/journal.pone.0240219)
Supplement: S2 Table — (DOCX) [file pone.0240219.s002.docx]

**S2 Table. Baseline characteristics of patients with endoscopic transpapillary gallbladder stenting (ETGBS) and endoscopic nasogallbladder drainage (ENGBD).**

|  | | | **ETGBS**  **(N = 83)** | | **ENGBD**  **(N = 72)** | ***P-* value** |
| --- | --- | --- | --- | --- | --- | --- |
| **Sex, male (%)** | | | 40 (48.2) | | 43 (59.7) | 0.151 |
| **Age, mean (SD)** | | | 78.94 (11.37) | | 65.90 (14.22) | 0.018 |
| **Cholecystectomy, n (%)** | | No | | 73 (88.0) | 12 (16.7) | < 0.001 |
|  | | Yes | | 10 (12.0) | 60 (83.3) |  |
| **ASA class, n (%)** | | I | | 0 | 15 (20.8) | < 0.001 |
|  | | II | | 11 (13.3) | 52 (72.2) |  |
|  | | III | | 70 (84.3) | 5 (6.9) |  |
|  | | IV | | 2 (2.4) | 0 |  |
| **Dementia, n (%)** | | | | 40 (48.2) | 2 (2.8) | < 0.001 |
| **Diagnosis** | Presence of CBD stone with ACC, n (%) | | | 66 (79.5) | 70 (97.2) | 0.001 |
|  | | ACC only, n (%) | | 17 (20.5) | 2 (2.8) | 0.001 |
| **Tokyo 2018** | | G2: moderate, n (%) | | 62 (74.7) | 1 (1.4) | < 0.001 |
| **Severity** | | G3: severe, n (%) | | 21 (25.3) | 71 (98.6) |  |
| **Visible cystic duct by cholangiography, n (%)** | | | 58 (69.9) | | 44 (61.1) | 0.251 |
| **CBD diameter (mm), mean (SD)** | | | 11.20 (3.7) | | 10.06 (3.5) | 0.472 |

*SD standard deviation, ASA American society of anesthesiologists, CBD common bile duct, ACC acute calculus cholecystitis, G grade.*
